# Seasonality and Grazing Exclusions Shape Bird Community Dynamics in West African Drylands

**DOI:** 10.1002/ece3.73607

**Published:** 2026-05-03

**Authors:** Alexandra Kuttnig, Ambroise N. Zongo, Ian Quintas, Liv Fritsche, Reto Spaar, Bakary Diakité, Franziska Kaguembèga‐Müller, Alain Jacot, Sabine B. Rumpf, Gabriel Marcacci

**Affiliations:** ^1^ Swiss Ornithological Institute Sempach Switzerland; ^2^ Department of Environmental Sciences University of Basel Basel Switzerland; ^3^ Association tiipaalga Ouagadougou Burkina Faso; ^4^ Department of Ecology and Evolution University of Lausanne Lausanne Switzerland; ^5^ newTree Bern Switzerland

**Keywords:** beta diversity, Burkina Faso, conservation, grazing, habitat restoration, land degradation, overgrazing, passive acoustic monitoring, Sahel

## Abstract

Dryland ecosystems are highly vulnerable to climate change and anthropogenic disturbances, leading to land degradation and biodiversity loss. Grazing management, including livestock exclusion, is widely used to restore vegetation, yet its effects on the spatiotemporal dynamics of species communities remain poorly understood. Here we studied variations in bird species composition (i.e., beta diversity)—a key indicator group of habitat restoration—between grazing exclusions and control sites over time in the seasonally dynamic Sahel region in Burkina Faso, West Africa. Bird communities were surveyed with passive acoustic monitoring in 25 small‐scale grazing exclusions and 50 control sites across two contrasting seasons. Seasonal effects were assessed by comparing the composition of bird species vocalization and vegetation parameters between dry and wet seasons. We conducted beta diversity analyses to measure the variation in species composition within and between grazing exclusions and control sites, and determined which vegetation parameters, derived from field inventories and remote sensing, best explained bird species spatial and seasonal turnover. The composition of bird species was more influenced by seasonal changes in the vegetation structure and productivity than by grazing exclusions. Moreover, bird communities within the grazing exclusions were more homogeneous than those in the control sites. Yet, grazing exclusions served as important refuges for birds during the dry season, presumably because they maintained a higher vegetation cover than the control sites throughout the year. Furthermore, high tree diversity and structurally complex vegetation promoted bird species turnover on a landscape‐scale. Due to its strong influence on bird communities, seasonality should be integrated into biodiversity monitoring and restoration planning of arid ecosystems. Although grazing exclusions were not the primary driver of community composition, they enhanced habitat heterogeneity, supporting regional bird biodiversity and providing refuges that mitigate the effects of overgrazing in this region of the Sahel.

## Graphical abstract

Seasonal changes, more than grazing exclusions, shape bird community composition in the Sahel region of Burkina Faso. However, grazing exclusions enhanced habitat heterogeneity, supporting regional bird biodiversity and providing seasonal refuges that mitigate the effects of overgrazing.

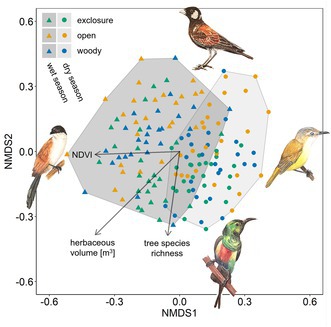

## 1 Introduction

Understanding the drivers of biodiversity across space and time is a central question in ecology and conservation biogeography. In arid regions, community compositions are shaped by strong seasonal dynamics of environmental conditions, marked by distinct wet and dry periods (Hiernaux et al. 1994; Walther 2016). This seasonality drives fluctuations in vegetation productivity and structure, which in turn influence habitat and resource availability for faunal communities (Olsen et al. 2015). In parallel, dryland ecosystems are severely threatened by anthropogenic pressures. Excessive livestock grazing and agricultural intensification in response to increasing food demands are major causes of land degradation and desertification (Maestre et al. 2022; Masson‐Delmotte et al. 2019). In the Sahel region of Sub‐Saharan Africa, grazing pressure becomes excessive when livestock are kept at high local densities and graze continuously throughout the year without seasonal resting periods, limiting vegetation recovery and leading to an overgrazed regime (Hiernaux et al. 2016). This negative trend in land condition is exacerbated by the effects of climate change, that is, reduced precipitation and increased frequency of drought events (Huang et al. 2016). The combination of human‐induced land degradation such as overgrazing and increased drought stress results in the loss of natural ecosystems and associated biodiversity (Maestre et al. 2022).

To combat land degradation and desertification, there is an urgent need to implement restoration measures. One widely applied restoration measure in the region is assisted natural regeneration through grazing exclusions (also known as “area exclosure” and called “mise en défens” in French; Maisharou et al. 2015; Reij et al. 2020). Grazing exclusions are fenced areas protected from livestock grazing to enable the natural regeneration of the vegetation, thus restoring degraded ecosystems (Chomba et al. 2020; Lohbeck et al. 2020). While their positive effects on the vegetation are well documented (Marcacci et al. 2025; Mathewos et al. 2023; Yaebiyo et al. 2024), evidence of their effectiveness for faunal communities remains limited (but see Barzan et al. 2021; Ortega‐Álvarez and Lindig‐Cisneros 2012). Moreover, most studies focused on the effect of habitat restoration on local species diversity (i.e., alpha diversity; Quintas et al. 2025), but it is unclear whether and how it can promote the turnover of species communities across sites and seasons, that is, beta diversity. Beta diversity describes the variation in community composition across space and/or time and reflects two underlying ecological processes: species replacement and differences in species richness (Baselga 2010, 2013b; Ponisio et al. 2016; Socolar et al. 2016). For example, habitat restoration measures such as grazing exclusions may not only increase species locally but also enhance landscape‐scale biodiversity by promoting spatial or temporal heterogeneity of habitats and resources (Ponisio et al. 2016). Furthermore, spatial and temporal gradients can interact to shape the spatiotemporal dynamics of species communities (Bennett et al. 2015; Karp et al. 2018). Beta diversity is therefore a critical component for evaluating the effectiveness of restoration measures to counter biotic homogenization and biodiversity loss.

In this study, we focus on bird communities. Birds provide multiple ecosystem services by acting as mobile links within and between ecosystems by dispersing seeds (de la Peña‐Domene et al. 2014) and pollinating plants (Medina‐Serrano et al. 2024), and contribute further to ecosystem health (Lundberg and Moberg 2003), for example by controlling pests (Ndang'ang'a et al. 2013; Tela et al. 2021). As mobile and habitat‐sensitive taxa, birds can moreover serve as reliable indicators of habitat restoration even in their early stages (Chowfin and Leslie 2020). Their distributions closely track environmental heterogeneity, resource availability, and vegetation structure, which can be directly influenced by both continuous grazing pressure and seasonal climatic fluctuations. In drylands globally, bird responses to land degradation vary: forest and shrub specialists often decline under simplified vegetation, while open‐ and edge‐habitat species may benefit, highlighting vegetation structure as a key driver of community composition and the potential for restoration to support diverse guilds (Macchi and Grau 2012; Maestre et al. 2016; Varun and Dutta 2020). Livestock grazing can have complex effects on bird communities, reducing abundance and species richness at high intensities, while moderate grazing can enhance bird diversity by maintaining diverse vegetation in grasslands (Barzan et al. 2021; Pillsbury et al. 2011). In addition, research from restored and tropical landscapes demonstrated that seasonally varying resource availability can strongly influence the composition and diversity of bird communities. This is critical, as also the benefits of restoration may consequently vary throughout the year, underscoring the importance of considering seasonal dynamics when planning and evaluating restoration efforts (Reid et al. 2014; Shi et al. 2024).

Most research investigating the effect of land degradation on birds in the Sahel zone focused on local bird diversity (Cresswell et al. 2007; Zwarts et al. 2009). A study that focused on the effects of grazing exclusions on alpha diversity (Quintas et al. 2025) demonstrated that they promote local bird species richness, especially in the dry season. However, land degradation and overgrazing can homogenize communities across larger spatial scales (Gossner et al. 2016; McKinney and Lockwood 1999; Socolar et al. 2016). Consequently, grazing exclusion can increase landscape‐scale diversity by allowing each site to develop its own distinct species composition, rather than all sites converging toward similar communities. Furthermore, grazing exclusions can increase both the spatial and temporal heterogeneity of bird community composition by reducing continuous high grazing pressure and thus allowing vegetation to recover. Despite the high seasonal dynamics of arid environments, most studies assessing the effectiveness of restoration measures do, however, not consider seasonal changes which can lead to biased conclusions about their actual impact. In addition, evidence from grazing exclusions in the Sahel remains overall limited compared to other dryland regions, such as the temperate and alpine grasslands of northern China, where grazing exclusion has been shown to enhance biomass, soil carbon, and vegetation structure over multi‐year timescales (Xiong et al. 2016).

Temporal beta diversity in dry ecosystems is particularly influenced by vegetation components that vary seasonally, especially herbaceous plants (Breman and Cisse 1977). For example, if only the rainy season is assessed, the limiting effect of scarce food resources on birds in the dry season is ignored, which results in misleading measures of overall biodiversity (Morel and Morel 1974). In addition, tree species vary in their phenology and vertical structure, and high tree diversity can thus provide for certain bird species a continuous food supply throughout the year (Zwarts et al. 2023a), resulting in higher bird diversity (Basile et al. 2016). To understand the influence of vegetation on bird community dynamics, it is thus necessary to link local habitat characteristics, such as tree diversity and vegetation structure, with spatial patterns and seasonal changes in bird communities.

To address these knowledge gaps, we investigated the impact of small‐scale grazing exclusions on bird species composition across space and time in Burkina Faso. Burkina Faso exemplifies the challenges of the Sahel region: a rapid human population growth, erratic rainfall, unsustainable land use, and desertification, threatening biodiversity and the livelihood of local human populations (Conagese 1999; Doso Jr 2014). These grazing exclusions were established and maintained with the aim of restoring degraded lands through passive regeneration, rather than planting, and to promote sustainable use of natural resources while improving the livelihoods of farming households. Compared to large‐scale exclusions, such small‐scale grazing exclusions are additionally more likely to enhance landscape‐scale heterogeneity. More specifically, we assessed variations in bird species composition between 25 grazing exclusions and paired control sites across the dry and the wet season using passive acoustic monitoring (PAM) to address four main research questions:

1. What are the individual and interactive effects of site type (grazing exclusion and control sites) and season (dry and wet) on bird communities? We expected bird communities to be shaped by both factors, but species turnover to be highest between site types as they benefit species with different needs.
2. Do spatiotemporal variations in community composition differ within each site type? We expected spatial turnover to be highest within grazing exclusions as these areas should have more complex and heterogeneous vegetation providing more available niches for bird species than the grazed surroundings (Karp et al. 2018). Vegetation and associated food resources are depleted in grazed areas during the dry season, making birds more dependent on grazing exclusion at this time (Quintas et al. 2025). Therefore, bird communities in grazing exclusions should exhibit less seasonal variations than those in control sites.
3. Do differences in bird species composition between grazing exclusions and control sites vary between the seasons? We expected that dissimilarities would be lower during the dry season than during the wet season, since open habitat bird species may rely more on grazing exclusions when resources are scarce.
4. Which parameters of the vegetation drive variations in bird species composition? The more similar the vegetation is, the more similar the bird communities. We therefore expected tree diversity and the structural heterogeneity of the vegetation to be among the main drivers of variation.

## 2 Methods

### 2.1 Study Area and Design

More than 400 grazing exclusions of approximately 3 ha each have been established across Burkina Faso since 2003 by the local population with the support of the organizations newTree (https://newtree.org) and tiipaalga (https://tiipaalga.org/). Hereafter, grazing exclusion denotes the general practice of excluding domestic livestock to protect the vegetation, while exclosure refers specifically to the permanently fenced study sites where this has been implemented. Notably, these permanent fences equally exclude wild grazers. For this study, we focused on the Plateau Central, Centre, and Centre‐Ouest regions, as these were the only areas where the security situation allowed fieldwork. Within these three regions, we selected exclosures that were between 5 and 16 years old to ensure that the vegetation had recovered (Marcacci et al. 2025), and that were located at least 1 km apart to reduce spatial autocorrelation.

The three regions are located in the transition zone between the wetter Sudanian savannahs in the southwest and the drier Sahel zone in the northeast. In addition to this location‐specific climatic gradient, these regions experience a strong seasonality: a short rainy season from June to September and a long dry season from October to May. During 1991–2020, mean monthly air temperatures reached maximum values of up to 40°C in April–May, while they did not fall below 25°C in December–January, and mean monthly precipitation sums ranged between 0 and 65 mm during dry season and 110 and 240 mm during the rainy season (World Bank Group 2023). These environmental conditions result in a seasonal Sudano‐Sahelian savannah woodland, historically dominated by thorny trees such as *Vachellia* (formerly *Acacia*), *Balanites*, and *Combretum* species, with *Vitellaria paradoxa* being particularly dominant, reflecting a mosaic of degraded savannahs, small‐scale agricultural fields, and villages (Marcacci et al. 2025). Exclosures are small but retain natural vegetation structure, providing typical conditions used by local bird communities. The avifauna includes typical common species from Sudano‐Sahelian savannahs, such as the common bulbul ( *Pycnonotus barbatus* ), the vinaceous dove ( *Streptopelia vinacea* ), and the yellow‐crowned gonolek ( *Laniarius barbarus* ), and no globally threatened species are present (Quintas et al. 2025).

To examine the effects of seasonality, two sampling rounds were conducted for both birds and vegetation. The first round took place from October to November 2022, just after the rainy season, when the vegetation was green and the available food resources for birds were at their maximum (hereafter referred to as wet season). The second round was conducted from March to April 2023 during the advanced dry season, when the vegetation in the landscape was severely reduced.

**FIGURE 1:**
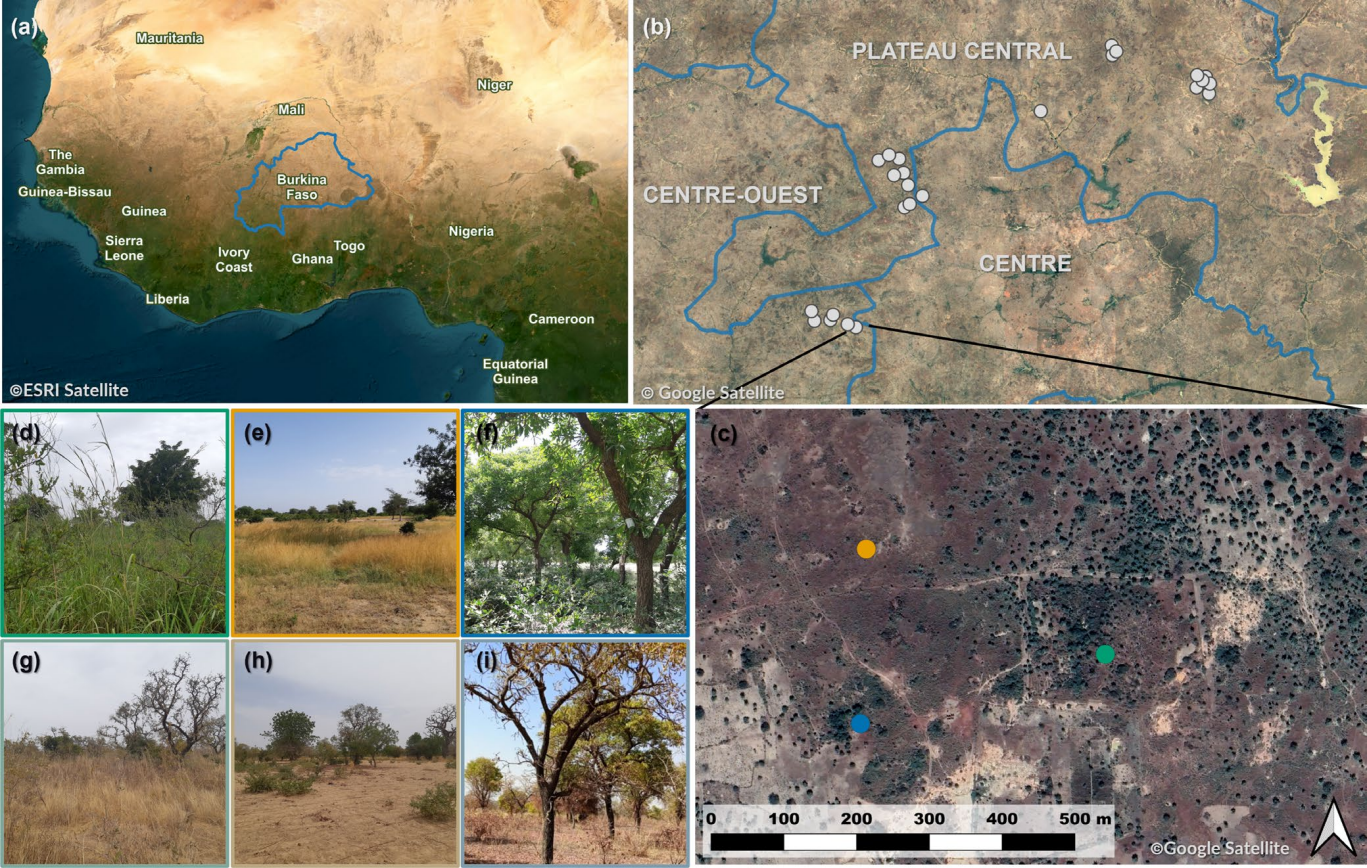
Study design map. (a) Location of Burkina Faso in West Africa. (b) Locations of the 25 landscape units are displayed in light gray (c) Example of a landscape unit with exclosure (green), open control (orange) and woody control (blue). Pictures illustrate exclosures (d, g), open controls (e, h) and woody controls (f, i) in the wet season (October) in the upper panels and in the dry season (March) in the lower panels. Pictures of panels taken by Gabriel Marcacci.

For each of the 25 exclosure sites, paired control sites were selected: one in a woody area and one in an open area of equal shape (i.e., squares) and size (i.e., 3 ha). In total, 75 sites were selected (Figure 1). Hereafter, an exclosure and its two paired control sites are referred to as a “landscape unit”. This design is comparable to that of similar studies in other regions (Karp et al. 2018) and allowed to assess community turnover at different scales. The two control sites were selected within a 500‐m radius around the centroid of each exclosure, while maintaining a minimum distance of 250 m from the exclosure boundary. This distance ensured the independence of acoustic recordings while keeping sites comparable in environmental conditions. The open controls were characterized by low tree density, whereas the woody controls were selected to match exclosures in terms of tree density, albeit with a lower vegetation regeneration potential as they remained accessible to domestic livestock. Control sites were first screened using aerial images and Normalized Difference Vegetation Index (NDVI) to assess vegetation characteristics, but finally selected in the field by visually comparing tree densities to exclosures. Thus, these controls allowed the examination of the impacts of maintained exclosures on bird community variation, both with and without considering similarities in tree density. Moreover, they enabled the analysis of the effects resulting from differences in local tree communities and seasonal variations in vegetation. All farmers and landowners gave their permission to access and conduct this study on their lands prior to conducting any research activities.

### 2.2 Bird Survey

Birds were recorded in 2022 and 2023 using passive acoustic monitoring (PAM) with autonomous recording units (ARU; AudioMoth Dev 1.2.0: Open Acoustic Devices 2023). One ARU was attached to a tree at 1.5 m above ground in the middle of each exclosure and control sites. The two sampling rounds covered the overwintering and passage season for migrant species as well as the breeding season for resident species, while maximizing contrasts in terms of environmental conditions. Recording began 30 min before dawn (start of civil twilight) and lasted 2 h (i.e., 90 min after dawn), including the peak activity of diurnal bird species and allowing for the recording of nocturnal species. The 2 h were recorded at a temporal resolution of 1‐min recordings every 6 min to increase detection rates (Metcalf et al. 2021), resulting in a total of 20 min of recordings per site and season, which is within the range of commonly used point count durations. To counteract variations in bird species composition caused by daily environmental fluctuations, all sites within a landscape unit were recorded on the same day. The limitation of PAM is that it captures species presence based on vocalizations, resulting in a potential bias for species that vocalize infrequently throughout the year. However, it has the advantage over other standard protocols (e.g., point counts) that sampling of data across large spatial and temporal scales at the same time is more efficient and standardized, and that detection rates of rare or nocturnal species are increased (Bobay et al. 2018; Darras et al. 2019; Sugai et al. 2018). This automated method was particularly advantageous in the study area because birds could be observed independently of the security situation and consequent difficulties of access.

Prior to the bird surveys, we verified that the detection ranges did not differ between exclosures and controls due to variation in sound propagation across different vegetation types following Darras et al. (2018). Although we acknowledge that detection distances vary between species, it is assumed that 95% of birds are detected within 40 m of an ARU in wooded areas (Darras et al. 2018). Each bird species in each sound recording was manually annotated and identified using the sound analysis software Raven Lite (Cornell Lab of Ornithology Store 2024), and the web application ecoSound‐web (Darras et al. 2020, 2022), which facilitates collaboration with sound files. Each of these annotations was also validated by an expert in African ornithology to minimize observer error or bias. Recordings of unknown species were annotated and validated by additional experts (see Acknowledgements). We identified all types of vocalizations (songs, calls, alarms, and contact calls), several of which are produced by both sexes in many Sahelian species; thus, detections reflect species presence rather than sex‐specific behavior. During data processing, we excluded detections that could not be assigned to species with sufficient confidence. This represented less than 1% of all detections (159 out of 18,232 bird detections). Furthermore, three species from the genus *Lamprotornis* and four species from the genus *Ploceus* were grouped together as these species could not always be reliably distinguished. Because home‐range sizes vary widely among Sahelian bird species and our acoustic data did not allow distinguishing whether individuals were foraging or holding territories inside or outside study sites, detections should be interpreted as site use rather than as evidence of breeding activity.

### 2.3 Vegetation

Exclosures were expected to show higher tree diversity, greater vertical structural complexity, denser herbaceous vegetation and lower seasonal variability in vegetation biomass due to livestock grazing exclusion. These a priori expectations guided our selection of vegetation metrics for analysis. To analyze the influence of local tree diversity on bird species composition, a tree inventory was conducted within a 30‐m radius around each ARU in October 2022. All tree species were identified, and their number of stems counted in each of four height strata corresponding to tree height (< 1, 1–2, 2–5, > 5 m). This provided not only tree information about diversity, that is, richness and abundance, but also about the vertical structure, that is, number of tree stems by strata (MacArthur and MacArthur 1961). To capture broader vegetation structure and its seasonal dynamics, we additionally quantified herbaceous volume and NDVI in both seasons independently. Percent cover and mean height of the herbaceous vegetation were estimated within a 10‐m radius around each ARU and seasonal herbaceous volume was then calculated as their product. NDVI values were obtained from five temporally distributed Sentinel‐2 satellite images at a resolution of 10 m within a 50‐m radius of each ARU for each season (i.e., temporally matching acoustic recordings; Pettorelli et al. 2005). From these values, we then calculated per site and season the maximum NDVI to reduce the impact of clouds and near‐surface dust, which cannot be removed from remote sensing images.

### 2.4 Statistical Analysis

All statistical analyses were conducted in R version 4.2.3 (R Core Team 2023).

#### 2.4.1 Effects of Site Type and Season on Bird Species Compositions

First, to investigate the relative effects of site type (exclosures, open and woody controls) and seasons (dry and wet) on overall bird species composition, we calculated pairwise beta diversity between all sites and seasons using the *metaMDS()* function from the *vegan* package (Oksanen et al. 2022) with *method = “bray”* which is equivalent to Sørensen dissimilarity index for presence‐absence data, that is, capturing the proportion of species not shared between communities. We used a non‐metric multidimensional scaling (NMDS) plot on the Sørensen dissimilarity metric to visually depict the effect of site type and seasons on bird species composition of each site. With the *gg_envfit()* function from the *vegan* package (Oksanen et al. 2022), we additionally evaluated the influence of the site‐specific vegetation parameters tree species richness, total number of tree stems across all four strata, NDVI and herbaceous volume on the observed Sørensen dissimilarities. Significant parameters (i.e., *p*‐value < 0.05) were retained and added to the NMDS plot. For assessing the statistical significance of site type and season effects, we conducted a permutational multivariate analysis of variance (PERMANOVA) using the *adonis2()* function from the *vegan* package (Oksanen et al. 2022), specifying an interaction between site type and season. To address potential spatial autocorrelation or site‐specific effects, we set landscape unit as a blocking factor. *p*‐values were computed on 999 permutations.

Additionally, we identified indicator bird species for site types and seasons using the *multipatt()* function from the *indicspecies* package (De Cáceres et al. 2010). This function defines indicator species by calculating the probability that the investigated site belongs to the target group (site types or season) when the species is found, as well as the probability of finding the species at sites that belong to the group. Thus, we identified which species were associated with a given season or site type (specificity) and evaluated the strength of these associations (sensitivity).

#### 2.4.2 Spatiotemporal Variations in Bird Species Composition Within and Between Site Types

First, to evaluate whether bird communities in exclosures exhibit greater spatial beta diversity than those in control sites, we compared pairwise Sørensen dissimilarities within each site type by combining bird communities from both seasons for each site (e.g., site number 1, site type 1 vs. site number 2, site type 1). These dissimilarities can result from species replacement or differences in species richness, representing distinct ecological processes (Baselga 2010, 2013b). Therefore, we partitioned the Sørensen dissimilarities into the two distinct components turnover and nestedness with the *betapart* package (Baselga and Orme 2012) to assess whether spatial beta diversity is more due to species replacement than due to differences in species richness. We assessed the mean distances of Sørensen dissimilarities and their turnover and nestedness components from each site to the centroid of all sites within the same site type (i.e., group centroids; Anderson et al. 2006) using the *betadisper()* function from the *vegan* package (Oksanen et al. 2022). We then estimated the differences of these centroid distances between site types with linear mixed‐effects models (LMMs) using the *lme4* package (Bates et al. 2015) for each aspect of beta diversity separately (i.e., three LMMs corresponding to dissimilarity, turnover, and nestedness), setting landscape unit as a random intercept. Subsequently, we used likelihood ratio tests on a Chi‐squared distribution to determine the significance of the obtained estimates (Zuur et al. 2009).

Second, seasonal variation of bird communities within each site type (i.e., temporal beta diversity) was equally assessed using Sørensen dissimilarity as well as its components turnover and nestedness but calculated as the dissimilarity between the two seasons within each site (e.g., site number 1, season 1 vs. site number 1, season 2). Whether temporal beta diversity was more pronounced in control sites than in exclosures was then tested with three separate LMMs, using one of the aspects of beta diversity as response variable, site type as fixed effect and landscape unit as random intercept, and a subsequent likelihood ratio test.

Third, we investigated whether differences in bird species compositions between site types were larger during the dry than during the wet season. To this end, we computed season‐specific Sørensen dissimilarity as well as its components turnover and nestedness between all three site types within a landscape unit (e.g., site 1, site type 1 vs. site 1, site type 2). Subsequently, we ran separate LMMs using one of the aspects of beta diversity as response variable, site type and season as interacting fixed effects and landscape unit as random intercept, and conducted a subsequent likelihood ratio test.

Because beta diversity can reflect random (or incomplete) sampling effects of individuals or species, it is important to account for these effects when comparing communities with different numbers of individuals or species. One common way to control for sampling effect in beta diversity metrics is the use of null models with randomization algorithms (e.g., see Chase et al. 2011). Which randomization algorithm is best and how to interpret the resulting corrected metrics is, however, subject to ongoing debate. Yet, McGlinn et al. (2025) recently proposed a new framework to control for sampling effects using rarefaction curves. We applied this new framework as implemented in the R‐package *mobr* (McGlinn et al. 2024) to all beta diversity analyses (see Appendix S1 for methodological details).

#### 2.4.3 Influence of Vegetation Parameters and Spatial Distance on Bird Species Turnover

We used distance‐decay models to analyze the influence of geographic distances, tree diversity and vegetation structure on bird species turnover within each site type, considering bird occurrences from both seasons. This method evaluates the change in compositional dissimilarity with increasing distance along chosen gradients between all possible site pairs (Baselga 2013a; Nekola and White 1999). We applied negative exponential distance decay models, fitted with the *decay.model()* function from the *betapart* package (Baselga and Orme 2012), which uses a generalized linear model (GLM) with a log link function and a Gaussian error distribution. Bird Sørensen turnover was used as the response variable to assess species replacement between sites and geographic distance, dissimilarity in tree species composition and dissimilarity in vegetation structure were used as predictor variables. Geographic distance, calculated with the *geodist()* function from the *geodist* package (Karney 2013), was included to evaluate dispersal limitations. Dissimilarity in tree species composition was calculated as Bray–Curtis dissimilarity (abundance data) with the *vegdist()* function from the *vegan* package (Oksanen et al. 2022) to assess the impact of trees, including their relative abundance and biomass contribution which reflects resource availability more accurately than tree species richness alone. Dissimilarity in vegetation structure was quantified as Gower dissimilarity using the *daisy()* function from the *cluster* package (Maechler et al. 2023). We weighted the numbers of tree individuals across all four strata equally and used herbaceous volume and the NDVI of both seasons to account for seasonal dynamics of the vegetation biomass and overall structural variation. The pairwise difference between all sites was calculated for each structural variable and then divided by the range of that variable to standardize it. The differences were then averaged across all variables to produce a single pairwise dissimilarity value. Thus, it captured different aspects of the vertical and horizontal structure in one metric.

To test whether turnover rates (i.e., the slope of decay) differ significantly between site types, we bootstrapped the slopes 1000 times with the *boot.coefs.decay()* function from the *betapart* package (Baselga and Orme 2012). We then compared the bootstrapped slopes between site types by calculating two‐tailed *p*‐values using the proportion of bootstrap values of one site type larger or smaller than the value of the other site type (Gómez‐Rodríguez and Baselga 2018).

## 3 Results

A total of 18,073 bird vocalizations from 88 species were annotated and identified (i.e., detection of a species within a 1‐min recording) of which 72% were found in all site types (Figure S2.1). We detected 82 species in exclosures, corresponding to 93% of all species. Open controls had 85% of all species, and woody controls had 82%. During the wet season, 83 species were detected, 84% of which were found in exclosures, 86% in open controls and 80% in woody controls. Notably, 95% of the 73 species detected during the dry season were found in exclosures, compared to 77% in open controls and 82% in woody controls. During the wet season, open controls hosted seven unique species, while exclosures and woody controls contained only three. By contrast, open controls and woody controls contained just one unique species during the dry season, whereas exclosures hosted eight. Overall, open controls and woody controls shared fewer species in common than either did with exclosures, and exclosures also had a higher total number of unique species.

### 3.1 Effects of Site Type and Season on Bird Species Composition

**FIGURE 2:**
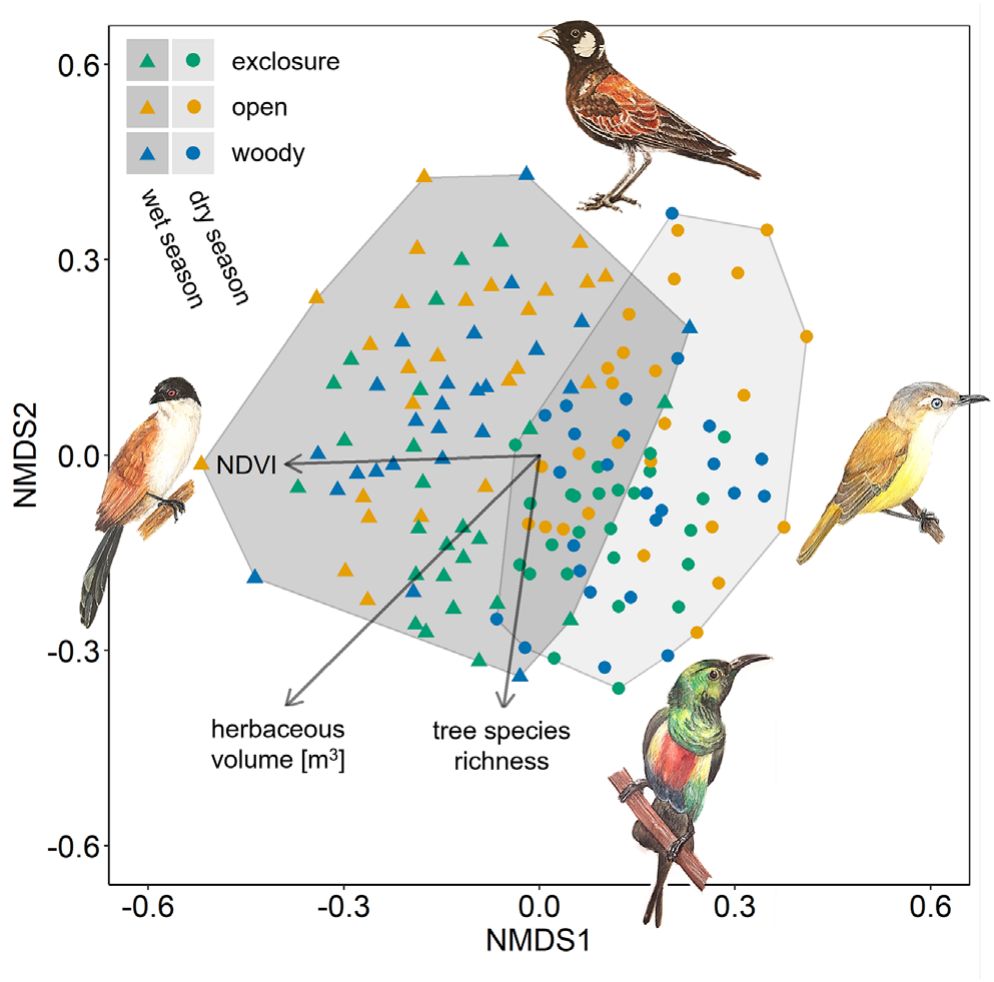
Effects of season and site type on bird species composition. Non‐metric multidimensional scaling (NMDS) of Sørensen dissimilarity in species composition represented by distances between all 75 sites in Burkina Faso for both seasons. Dots are sites in dry season, triangles sites in wet season, and point colors depict site types. Arrows indicate the direction of the maximum variable contribution to local max. NDVI (measured within 50‐m radius), herbaceous volume and tree species richness. The clusters depict the clear split between the seasons. The Senegal Coucal *Centropus senegalensis* (left) is the indicator bird species of wet season communities, while the Senegal Eremomela *Eremomela pusilla* (right) represents the dry season communities. The Beautiful Sunbird *Cinnyris pulchellus* (bottom) is associated with exclosure, and the Chestnut‐backed Sparrow‐Lark *Eremopterix leucotis* (top) signifies grazed sites, mainly open controls. Drawings by Alexandra Kuttnig.

Variation in bird communities along the first NMDS axis was mainly linked to seasonal changes and was correlated with NDVI (Figure 2). The variation along the second NMDS axis was influenced by herbaceous volume and tree species richness. Although exclosures tended to have higher tree species richness and a higher herbaceous volume than the controls, the NMDS ordination represented the Sørensen dissimilarities of bird communities only poorly (Stress value = 0.299). Nevertheless, the PERMANOVA highlighted the significant effects of both season (*F* = 18.535, *p*‐value < 0.001) and site type (*F* = 4.616, *p*‐value < 0.001) on differences in bird species composition. Season had twice the explanatory power of site type (*R* ^2^ = 0.107 vs. *R* ^2^ = 0.053), and there was no interactive effect between season and site type (*F* = 0.964, *p*‐value = 0.245). Notably, six bird species were significant indicators of exclosures, but only three were indicators of open controls and none of woody controls (Table S2.1).

### 3.2 Spatiotemporal Variations in Bird Species Composition Within and Between Site Types

**FIGURE 3:**
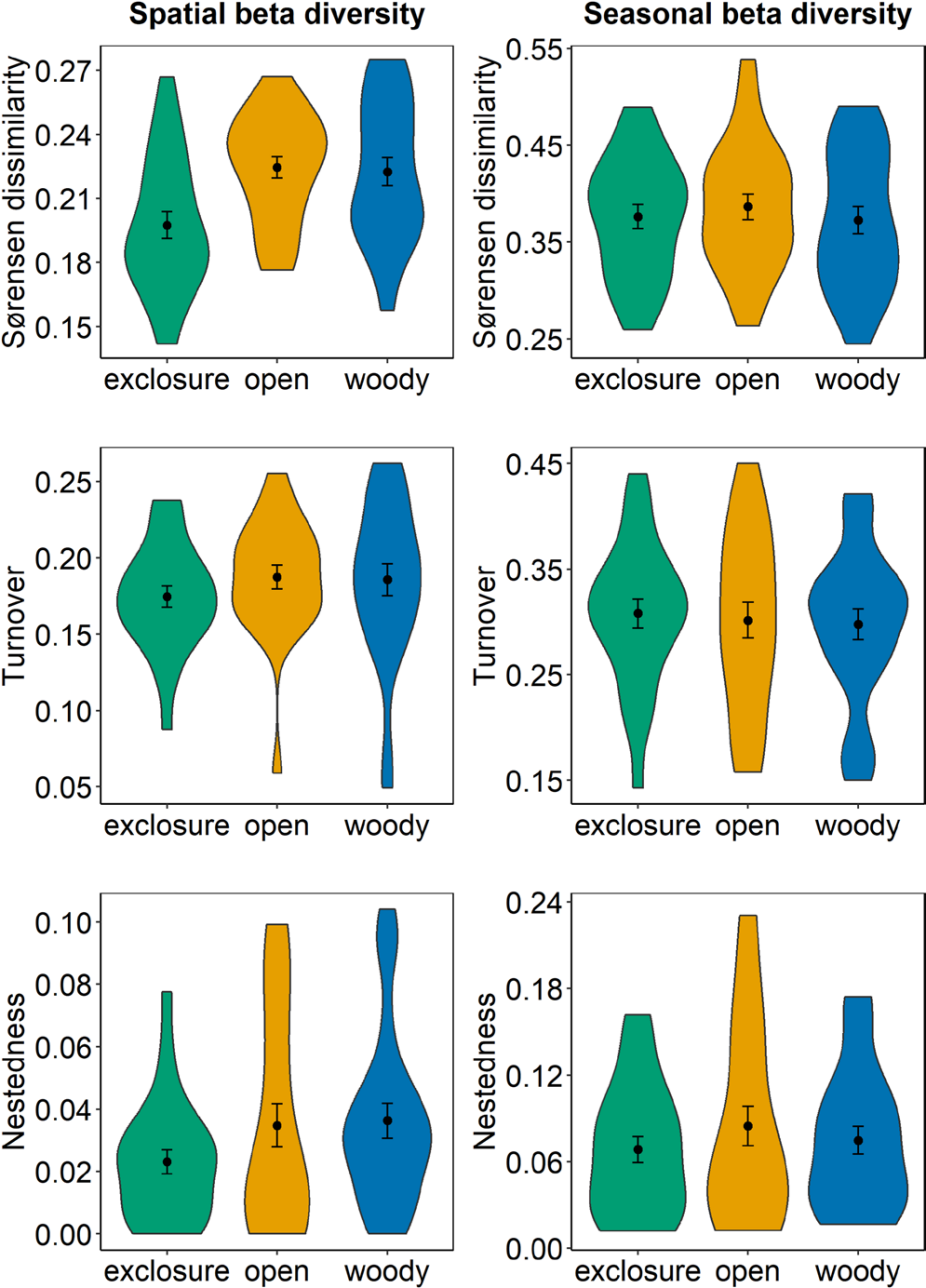
Spatial and seasonal variations of bird communities in Burkina Faso within site types. Beta diversity was calculated as Sørensen dissimilarity and its components species turnover and nestedness. Spatial beta diversity was calculated as mean distances of the 75 sites to the group centroids of the respective site type. Seasonal beta diversity was calculated within the 75 sites between seasons. The violin plots depict the distributions of the beta diversity metrics, the dots the mean beta diversity within site types, and the error bars the standard errors derived from linear mixed‐effects models (LMMs).

Spatial Sørensen dissimilarities within exclosures were significantly smaller than within both control areas (*χ* ^2^ = 16.645; *p*‐value < 0.001; Figure 3; Table S2.2), but bird species turnover and nestedness did not differ significantly between site types (turnover: *χ* ^2^ = 1.465; *p*‐value = 0.481; nestedness: *χ* ^2^ = 3.417; *p*‐value = 0.181; Table S2.2). No significant differences based on site types were found for seasonal beta diversity (Sørensen: *χ* ^2^ = 0.603; *p*‐value = 0.74; turnover: *χ* ^2^ = 0.245; *p*‐value = 0.885; nestedness: *χ* ^2^ = 1.193; *p*‐value = 0.551; Figure 3; Table S2.3). Overall, both seasonal and spatial species turnover were higher than nestedness in all site types (Figure 3; Tables S2.2 and S2.3).

**FIGURE 4:**
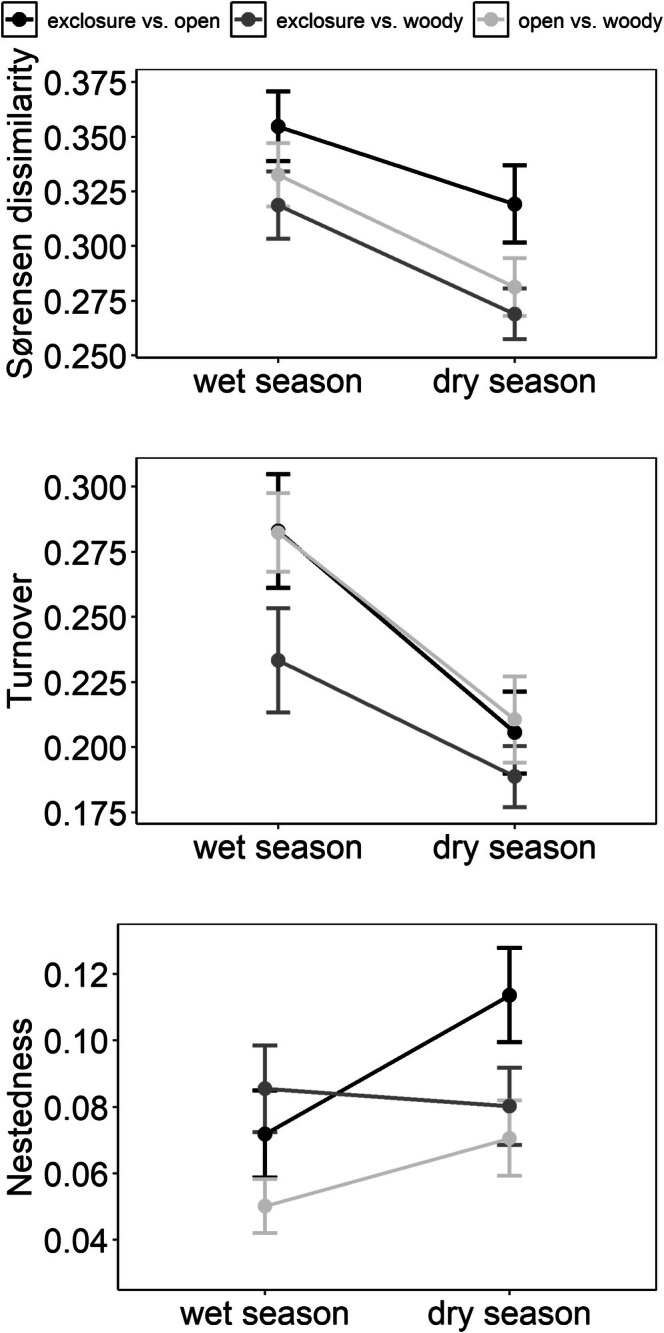
Variations of bird communities in Burkina Faso between site types and seasons within 25 landscape units. Beta diversities were calculated as Sørensen dissimilarity and its components species turnover and nestedness for all three different relationships of site types (coloring). The dots show the mean beta diversity within landscape units between site types per season and the error bars represent the standard error derived from linear mixed‐effects models (LMMs). The lines depict the seasonal change of the beta diversity metrics.

Overall, bird Sørensen dissimilarity and bird species turnover between site types within a landscape unit were higher in the wet season than in the dry season (Figure 4; Table S2.4). Nestedness between exclosures and open controls was higher in the dry season than in the wet season (*χ* ^2^ = 4.643; *p*‐value = 0.031), but there was no seasonal difference between exclosures and woody controls.

Beta‐diversity analyses accounting for sampling effects produced qualitatively the same patterns, confirming that the results were robust and not affected by random or incomplete sampling (see Appendix S1).

### 3.3 Influence of Vegetation Parameters and Spatial Distance on Bird Species Turnover

**FIGURE 5:**
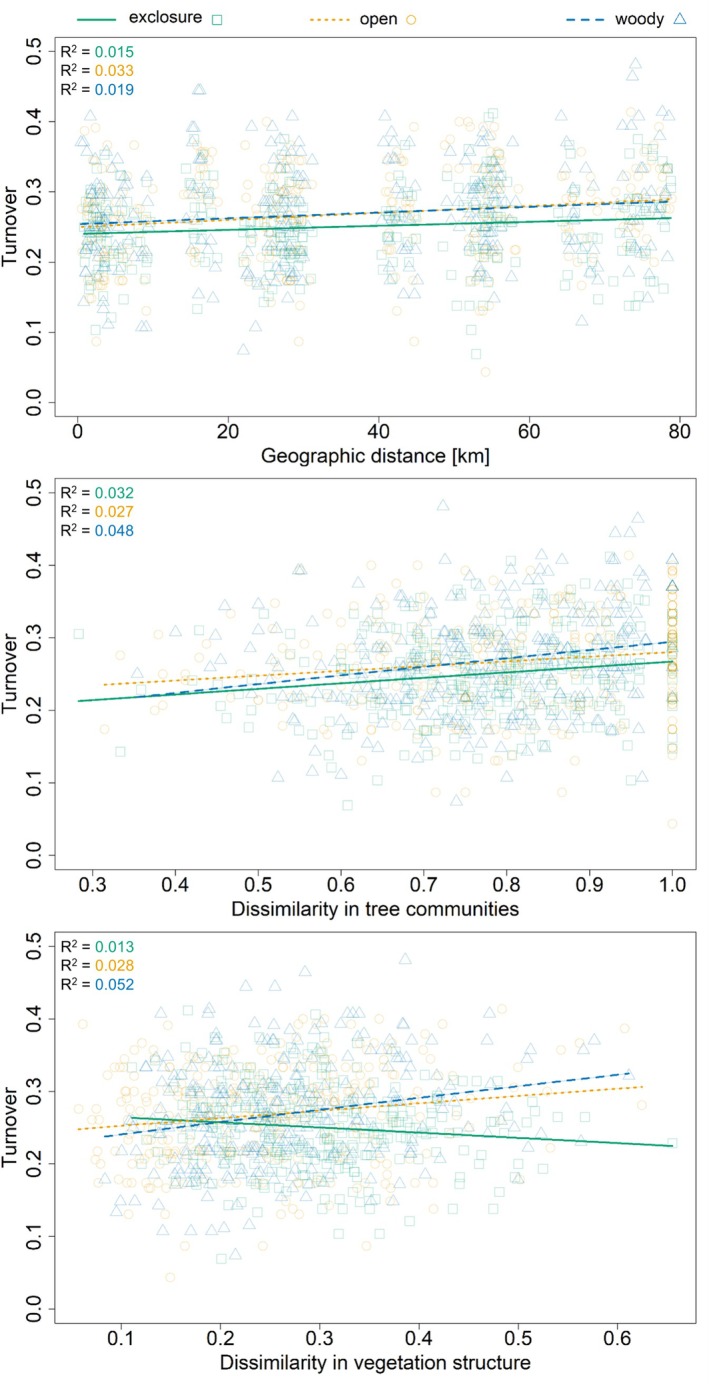
Influence of vegetation on bird species turnover. Distance–decay plots of bird community turnover rate (Sørensen) in Burkina Faso with increasing geographic distance, dissimilarities in tree communities (Bray Curtis), and differences in vegetation structure (Gower dissimilarity) between pairs of 75 sites within site types (color, shape). The lines represent decay models' relationship. The dots depict all pairwise comparisons.

With increasing geographic distance and increasing tree diversity dissimilarity, bird communities became more dissimilar (Figure 5). These relationships were consistent across all site types. For exclosures and woody controls, bird turnover was better explained by local differences in tree species composition (exclosure: pseudo *R* ^2^ = 0.032, *p*‐value = 0.045; woody: pseudo *R* ^2^ = 0.048, *p*‐value = 0.012) than by dispersal limitations (exclosure: pseudo *R* ^2^ = 0.015, *p*‐value = 0.059; woody: pseudo *R* ^2^ = 0.019, *p*‐value = 0.042). In open controls, geographic distance explained bird turnover slightly more (pseudo *R* ^2^ = 0.033; *p*‐value = 0.004) than tree diversity (pseudo *R* ^2^ = 0.027; *p*‐value = 0.046). The highest vegetation structure dissimilarity of site types was found within exclosures (Figure S2.2), indicating greater heterogeneity of vegetation structure as well as preservation of vegetation biomass during the dry season. However, bird turnover within exclosures and open control did not increase with this vegetation structure dissimilarity (exclosure: pseudo *R* ^2^ = 0.013, *p*‐value = 0.218; open: pseudo *R* ^2^ = 0.028, *p*‐value = 0.089), unlike in the woody controls (pseudo *R* ^2^ = 0.052; *p*‐value = 0.039). Bird communities in exclosures responded the least strongly to changes in vegetation structure, being distinct from both open (bootstrapped *p*‐value < 0.001) and woody (bootstrapped *p*‐value < 0.001) controls.

## 4 Discussion

Detected vocalizations revealed that grazing exclusion positively impacted landscape‐scale bird diversity by providing high tree diversity and complex vegetation structure. This was particularly evident during the dry season when birds congregated in areas with preserved vegetation, resulting in an even stronger influence of vegetation seasonality than of grazing exclusions on bird species composition. Our results thus highlight the need to incorporate seasonality when planning and evaluating restoration efforts in semi‐arid ecosystems such as the Sahel.

### 4.1 Effects of Site Type and Season on Bird Species Composition

Both site type and season influenced bird species composition. However, turnover in the bird community was more pronounced between the dry and the wet season than between exclosures and the two control areas. This result contrasts with a similar study conducted in Costa Rica that found a stronger effect of land use than of precipitation on bird community composition (Karp et al. 2018). One possible explanation is the differing magnitude of investigated land use practices and precipitation seasonality. In Costa Rica, contrasting land use types were investigated (agriculture versus tropical forest), whereas all our site types were located within a semi‐arid savanna landscape and differences in land use were thus likely more subtle. By contrast, the seasonal fluctuations of precipitation are much more pronounced in the Sahel than in Costa Rica. This is reflected by strong seasonal fluctuations in plant biomass in general (i.e., NDVI) and in the understory (i.e., herbaceous volume). Although variation in NDVI persisted between site types across seasons and exclosures sustained the highest NDVI throughout the year, NDVI decreased strongly across all site types as the dry season progressed. In addition, this temporal decline was very similar between site types (57% in exclosures, 58% in woody controls, and 61% in open controls; Figure S2.2). Thus, seasonal changes in vegetation exerted a stronger influence on bird communities than differences between site types. Nevertheless, although the NDVI of different site types influences bird communities (Beresford et al. 2019), it only indicates photosynthetic‐active biomass and can, by default, not inform about the understory or the diversity and structure of the vegetation, which are likely to be more decisive for bird species community composition as they reflect the availability of different habitats and resources.

Indeed, differences in bird communities attributed to site types were more influenced by tree diversity and herbaceous volume than by NDVI. This indicates a significant influence of structural heterogeneity on bird communities (Culbert et al. 2013) but might also imply that birds depend on specific tree species (Zwarts et al. 2023b). Heterogeneous woody habitats are known to promote bird diversity by offering many ecological niches (MacArthur and MacArthur 1961), and indeed, tree diversity appeared to promote bird species turnover across all sites in our study. However, structural vegetation variability only increased bird turnover in the grazed controls, while bird communities in exclosures showed lower turnover rates despite high vegetation complexity. Grazed controls experienced stronger seasonal changes in herbs and shrubs, which may create temporal niches for bird species associated with non‐tree components, whereas exclosures remained structurally more stable in their understory.

In addition, our results differed from those found in Costa Rica in another aspect (Karp et al. 2018). The effects of season and site type on bird community turnover were independent rather than interactive. Thus, the type of site did not influence the seasonal turnover of bird communities. This independence may be attributed to the decoupling of seasonal variations in bird species occurrence from specific habitat preferences (Bennett et al. 2015). It is indeed known that the climatic conditions in specific seasons determine the habitat preferences of certain bird species (Frishkoff et al. 2016). This assumption is corroborated by the lack of differences in seasonal beta diversity between site types. For example, certain birds like the Senegal Eremomela ( *Eremomela pusilla* ) were commonly found across all site types but only during the dry season, while others like the Senegal Coucal ( *Centropus senegalensis* ) were more prevalent during the wet season. Conversely, some bird species exhibit preferences for specific habitat types throughout the year, potentially because these habitats offer distinct resources. In exclosures, species such as the Beautiful Sunbird ( *Cinnyris pulchellus* ), a nectarivore, and the Singing Cisticola ( *Cisticola cantans* ), an insectivore, were prominent, while the Chestnut‐backed Sparrow‐Lark ( *Eremopterix leucotis* ), a granivorous species that prefers open grasslands, was associated with open sites.

The strong seasonal variation of bird communities detected here might, in part, reflect limitations of PAM that detects bird species by their vocal activity which can vary seasonally (e.g., during the breeding season). In the Sahel, the wet season overlaps with the main breeding period of many resident species, often resulting in elevated song output, whereas some species sing less during the dry season or while molting. Additionally, some species may breed year‐round or show individual variation in singing, making it impossible to distinguish whether a bird is truly absent or simply less vocal. For example, the Black‐billed Wood‐Dove ( *Turtur abyssinicus* ) was an indicator of wet season communities, but this is more likely due to its lower vocal activity during the dry season (personal observation). In other words, it is not possible to distinguish whether a bird is truly absent or is not detected because it is less vocal or silent. Therefore, we recommend that acoustic monitoring data are supplemented with point count data or other visual observations to control for variations in vocal activity, particularly if the focus is on seasonal effects on bird communities. If visual corroborations are not feasible, models that account for variations in detection probability across seasons could offer a statistical workaround. For example, hierarchical dynamic (or multi‐seasons) multispecies occupancy models (Kéry and Royle 2020) accommodate variations in detection probability among individual species, thereby encompassing seasonal fluctuations (Royle et al. 2005; Royle and Kéry 2007). Although our study lacks this aspect (partly due to the security situation in our study region), the differences between site types and the seasonal changes in these differences could be assessed as this limitation does not affect such relative relationships. Nevertheless, the strong seasonal influence on variation in bird communities detected emphasizes the need to incorporate temporal beta diversity when assessing the effectiveness of conservation measures for biodiversity.

### 4.2 Spatiotemporal Variations in Bird Composition Within and Between Site Types

Contrary to our expectations, spatial bird species turnover was slightly lower among exclosures than in both controls, and this difference persisted when accounting for sampling effects (e.g., differences in the number of species and number of detections; Figure S1). This suggests that bird communities in exclosures are spatially more homogenous than those in controls. On the one hand, this could be driven by a process of additive homogenization with formerly rare or absent species becoming widespread (e.g., Green‐winged Pytilia *Pytilia melba* ; see Socolar et al. 2016). Alternatively, it could indicate that exclosures are well‐connected, resource‐rich but rather homogenous habitats, while control sites are overall more heterogenous, thus promoting bird species turnover. This interpretation is consistent with the idea that grazing exclusions provide optimal habitat and foraging conditions for many bird species by increasing the complexity of resources, environmental gradients and available niche space (Stein et al. 2014). However, although exclosures supported high vegetation complexity, their bird communities were relatively uniform. This suggests that the enhanced habitat quality supports many species simultaneously but limits spatial differentiation among them. In contrast, bird communities in control sites appeared to track variability in vegetation structure more directly, with higher turnover observed in more structurally diverse patches. These findings are consistent with previous studies from the Ethiopian Highlands and Costa Rica (Karp et al. 2018; Marcacci et al. 2022), which demonstrated that vegetation heterogeneity drives regional species turnover, whereas overall tree cover only becomes limiting below species‐specific thresholds (Buchanan et al. 2020). Another possible explanation involves the nonlinear relationship between grazing intensity and bird diversity. Intermediate grazing can result in higher overall bird diversity than very high but also very low grazing intensities by increasing environmental heterogeneity (Barzan et al. 2021). In addition, the type of livestock can further influence the effect of grazing intensity on bird diversity. For example, cattle often have a more pronounced effect than sheep or mixed grazers (Boyce et al. 2021). This underscores the need to consider grazing intensity and grazer identity when evaluating the effect of livestock presence on biodiversity.

Interestingly, spatial turnover between site types was higher during the wet season than during the dry season, a pattern also observed in bird communities along precipitation gradients in Costa Rica (Karp et al. 2018). At first glance, this may seem counterintuitive, given that vegetation differences between site types were more pronounced during the dry season, which would suggest greater community differentiation. However, during the dry season, bird communities in open sites increasingly resembled those in exclosures, as many species move to areas with denser vegetation and more resources. This convergence was reflected in increased nestedness (the richness component of beta diversity), indicating that open‐control communities became subsets of those in exclosures. When vegetation is scarce, birds aggregate in resource‐rich exclosures, reducing community differentiation across site types. This result suggests that exclosures, although small in size, can affect bird communities on a larger scale. This highlights the importance of grazing exclusions as refuges during the dry season and underscores their conservation value in maintaining bird diversity in arid regions like the Sahel. Despite this seasonal convergence, spatial turnover between site types remained highest between exclosures and open grasslands. This is to be expected, given that the high vegetation cover in exclosures supports woodland‐associated species such as the Gray‐headed Bushshrike ( *Malaconotus blanchoti* ), while excluding open‐habitat specialists such as the Chestnut‐backed Sparrow‐Lark ( *Eremopterix leucotis* ; Quintas et al. 2025). It is well established that species such as larks avoid dense, complex habitats such as exclosures, even during the dry season (Marcacci et al. 2020). This demonstrates a limitation of grazing exclusions: while they promote species associated with woody vegetation, they are less effective at conserving open‐habitat specialists. Therefore, complementary conservation measures targeting open areas that harbor unique species are essential to sustain overall avian diversity. In this context, small‐scale grazing exclusions contribute to landscape heterogeneity but should be integrated within a mosaic of synergistic strategies that also include areas with moderate grazing intensities to support the full spectrum of habitat preferences of bird species.

## 5 Conclusion

Our study demonstrated that seasonal changes in vegetation had a greater influence on the composition of bird communities in the Sahel region than differences in site types (degraded vs. restored). However, excluding livestock grazing allows the native vegetation to recover naturally, enhancing landscape‐scale bird diversity, particularly during the dry season, by providing structurally complex, resource‐rich habitats that serve as ecological refuges. Tree diversity and vegetation structure emerged as key predictors of bird species turnover, emphasizing the importance of maintaining habitat heterogeneity to support avian biodiversity. These findings underline the critical importance of considering seasonal dynamics when assessing the impact of habitat restoration on species communities. However, due to seasonal biases in bird vocal activity affecting PAM, future studies should either supplement acoustic data with visual surveys or statistically adjust for detection variability to improve seasonal turnover inferences. While our results demonstrate clear biodiversity benefits of excluding grazing in this overgrazed region, the long‐term ecological role of moderate grazing in Sahelian savannas remains insufficiently understood. Future research should therefore evaluate whether controlled, moderate grazing could help to maintain habitat heterogeneity without compromising vegetation recovery, a question that is particularly relevant given the strong pressure on land and the socio‐economic dependence on livestock in the Sahel region. Strategies aimed at restoring structurally and compositionally diverse habitats while including the socio‐economic needs of the local human population will thus be essential in combatting land degradation and sustaining biodiversity in the light of increasing climatic variability.

## Author Contributions

**Alexandra Kuttnig:** data curation (equal), formal analysis (lead), investigation (equal), methodology (equal), visualization (lead), writing – original draft (lead). **Ambroise N. Zongo:** investigation (lead), methodology (supporting), writing – review and editing (supporting). **Ian Quintas:** data curation (equal), investigation (equal), writing – review and editing (supporting). **Liv Fritsche:** data curation (supporting), investigation (supporting), writing – review and editing (supporting). **Reto Spaar:** conceptualization (supporting), project administration (equal), writing – review and editing (supporting). **Bakary Diakité:** conceptualization (supporting), project administration (supporting). **Franziska Kaguembèga‐Müller:** conceptualization (supporting), project administration (supporting), writing – review and editing (supporting). **Alain Jacot:** conceptualization (equal), methodology (supporting), project administration (equal), supervision (supporting), writing – review and editing (supporting). **Sabine B. Rumpf:** conceptualization (equal), methodology (equal), supervision (equal), writing – review and editing (equal). **Gabriel Marcacci:** conceptualization (equal), investigation (equal), methodology (equal), project administration (equal), supervision (equal), writing – review and editing (equal).

## Funding

The authors have nothing to report.

## Conflicts of Interest

The authors declare no conflicts of interest.

## Supporting information


**Appendix S1:** ece373607‐sup‐0001‐Supinfo01.docx.

## Data Availability

All data supporting the results of this study are made available online on the vogelwarte.ch Open Repository and Archive in Zenodo: https://doi.org/10.5281/zenodo.15721271.
