# Supplementary material for: Seasonality and Grazing Exclusions Shape Bird Community Dynamics in West African Drylands
Source: Ecol Evol. 2026 May 3;16(5):e73607. doi: 10.1002/ece3.73607 (PMC13135892; doi:10.1002/ece3.73607)
Supplement: Supplementary file 1 — Appendix S1: ece373607‐sup‐0001‐Supinfo01.docx. [file ECE3-16-e73607-s001.docx]

**Appendix S1**

**Controlling for sampling effects**

As beta diversity can reflect random (or incomplete) sampling effects of individuals or species, it is important to account for these effects when comparing communities with different number of individuals or species. We therefore applied a recently developed framework implemented in the R-package *mobr* (McGlinn et al., 2024) to factor out the effects of incomplete sampling (McGlinn et al., 2025). First, we created a rarefaction curve for the sum of all sites within each site type (gamma diversity; Figure S1.1a). Because it is not possible to directly measure species abundances with passive acoustic monitoring, we used the number of detections instead of number of individuals. In our study, a detection corresponds to a species being present within a 1-minute recording. The number of detections for each species per site and season can be seen as a rough proxy for species relative abundances. Moreover, as for the number of individuals, we can expect a positive relationship between the number of detections and the number of species. Coming back to the rarefaction curve, it shows the expected number of species from a random sample of detections from all sites within each site type. Next, we calculated the rarefaction curves for each site and averaged them across each site type (alpha diversity, Figure S1.1b). Alpha-scale rarefaction curves are estimated up to the number of detections that provide a specific target level of sampling completeness, i.e. how close the gamma curve has reached a hypothetical asymptote which is estimated with the R function *mobr::* *calc_C_target.* Finally, we calculated coverage-based beta diversity for each site type as the ratio between gamma diversity and alpha diversity for a target number of detections that meets a particular level of sampling completeness with the R function *mobr::calc_beta_div()*. We calculated spatial (Figure S1.1c), as well as temporal (Figure S1.1d) coverage-based beta diversity.

**References**

McGlinn, D., Xiao, X., McGill, B., May, F., Engel, T., Oliver, C., Blowes, S., Knight, T., Purschke, O.,Gotelli, N. and Chase, J. 2024. mobr: Measurement of Biodiversity. R package version 3.0.0. <https://CRAN.R-project.org/package=mobr>

McGlinn, D.J., S.A. Blowes, M. Dornelas, T. Engel, I.S. Martins, H. Shimadzu, N.J. Gotelli, A. Magurran, B.J. McGill, and J.M. Chase. 2025. Disentangling non-random structure from random placement when estimating β-diversity through space or time. Ecosphere. e70061. <https://doi.org/10.1002/ecs2.7006114>

**Figure S1.1.** **Coverage-based beta diversity:** (a) gamma-scale rarefaction curves for each site type, with inset (b) showing average alpha-scale rarefaction curves for each site type; (b) mean spatial and (c) temporal coverage-based beta diversity. Points show means and error bars 95% quantiles for each site type. Means and quantiles were calculated using leave-one-out Jackknife resampling.


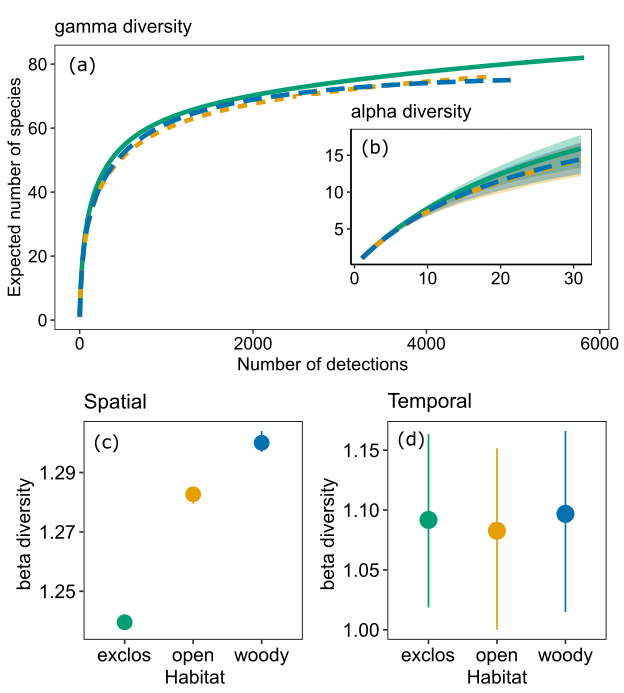


**Appendix 2**


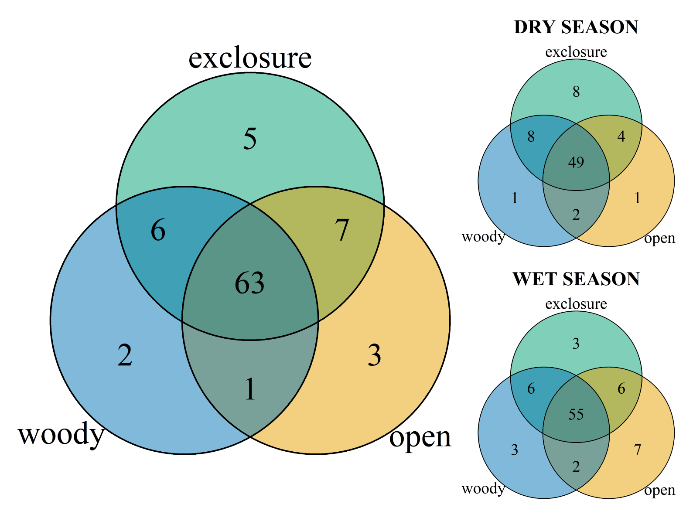


**Figure S2.1.** **Bird species shared between site types.** Venn diagram on the left shows bird species shared between site types with both seasons pooled together and diagrams on the right for the individual seasons. The number of bird species is shown in the circles.


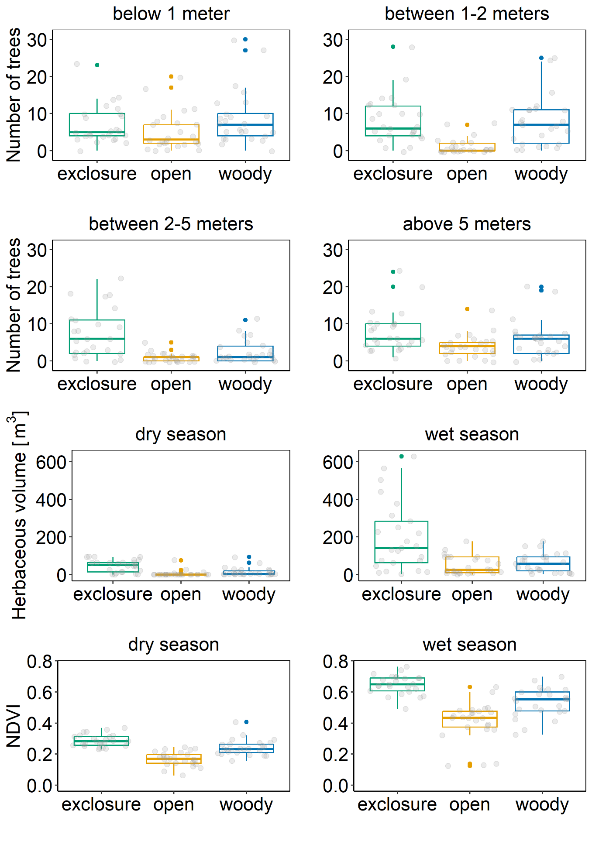


**Figure S2.2. Vegetation parameters measured in the field or with remote sensing.** Number of tree individuals per strata, herbaceous volume in m^3^ for both seasons and maximum NDVI within 50-meter radius for both seasons. Gray dots display the raw data of 25 sites per site type and season. Boxplots show the median, interquartile range (box), the smallest and largest values no further than 1.5 the interquartile range (whiskers) and more extreme values (dots) of the raw data.

| **Table S2.1. List of indicator bird species associated to season and to site type.** Multilevel pattern analyses conducted with significance level 0.1 and 999 permutations. Indicator value indices (Indval) are square rooted and defined by A specificity to group and B sensitivity of association to group. Significance codes: 0.001 ***, 0.01 **, 0.05 *, 0.1 . | | | | | | |
| --- | --- | --- | --- | --- | --- | --- |
| **Season** | **Indicator species** | **A** | **B** | **Indval** | **p-value** | |
| **Dry season** | *Prinia subflava* | 0.7245 | 0.9467 | 0.828 | 0.001 | *** |
|  | *Ploceus* sp. | 0.7273 | 0.8533 | 0.788 | 0.001 | *** |
|  | *Eremomela pusilla* | 0.8298 | 0.52 | 0.657 | 0.001 | *** |
|  | *Cinnyris pulchellus* | 0.7209 | 0.4133 | 0.546 | 0.002 | ** |
|  | *Cossypha niveicapilla* | 0.8636 | 0.2533 | 0.468 | 0.001 | *** |
|  | *Estrilda troglodytes* | 0.7619 | 0.2133 | 0.403 | 0.018 | * |
|  | *Sylvietta brachyura* | 0.75 | 0.2 | 0.387 | 0.026 | * |
|  | *Cercotrichas galactotes* | 0.75 | 0.16 | 0.346 | 0.063 | . |
|  | Sturnidae sp. | 0.7857 | 0.1467 | 0.339 | 0.043 | * |
|  | *Pytilia melba* | 0.7692 | 0.1333 | 0.32 | 0.075 | . |
| **Wet season** | *Turtur abyssinicus* | 0.732 | 0.9467 | 0.832 | 0.001 | *** |
|  | *Centropus senegalensis* | 0.7895 | 0.6 | 0.688 | 0.001 | *** |
|  | *Eremopterix leucotis* | 0.8824 | 0.2 | 0.42 | 0.002 | ** |
|  | *Phoenicurus phoenicurus* | 1 | 0.16 | 0.4 | 0.001 | *** |
|  | *Cisticola juncidis* | 1 | 0.1333 | 0.365 | 0.003 | ** |
|  | *Oriolus auratus* | 0.7647 | 0.1733 | 0.364 | 0.025 | * |
|  | *Ptilopachus petrosus* | 1 | 0.1067 | 0.327 | 0.005 | ** |
|  |  |  |  |  |  |  |
| **Site type** | **Indicator species** | **A** | **B** | **Indval** | **p-value** | |
| **Exclosure** | *Cinnyris pulchellus* | 0.579 | 0.88 | 0.714 | 0.004 | ** |
|  | *Cisticola cantans* | 0.583 | 0.84 | 0.7 | 0.009 | ** |
|  | *Euplectes franciscanus* | 0.654 | 0.68 | 0.667 | 0.001 | *** |
|  | *Glaucestrilda caerulescens* | 0.625 | 0.6 | 0.612 | 0.005 | ** |
|  | *Luscinia megarhynchos* | 0.909 | 0.4 | 0.603 | 0.001 | *** |
|  | *Pytilia melba* | 0.769 | 0.4 | 0.555 | 0.001 | *** |
| **Open** | *Vanellus tectus* | 0.645 | 0.8 | 0.718 | 0.001 | *** |
|  | *Eremopterix leucotis* | 0.688 | 0.44 | 0.55 | 0.01 | ** |
|  | *Galerida cristata* | 0.667 | 0.32 | 0.462 | 0.04 | * |
| **Exclosure + woody** | *Phylloscopus trochilus* | 0.917 | 0.44 | 0.635 | 0.008 | ** |
|  | *Estrilda troglodytes* | 0.889 | 0.32 | 0.533 | 0.064 | . |
| **Open + woody** | *Gymnoris dentata* | 1 | 0.18 | 0.424 | 0.068 | . |

| **Table S2.2. Differences in spatial beta diversity within site types.** Linear mixed-effects models were fitted by REML (restricted maximum likelihood). χ² and p-values are calculated from comparing nested models (with and without site type) via likelihood ratio tests. | | | | | | | |
| --- | --- | --- | --- | --- | --- | --- | --- |
| **Beta diversity metrics** |  | **Site type** | **Estimate** | **SE** | **X²** | **p-value** | |
| Sørensen |  | exclosure | 0.197 | 0.006 |  |  |  |
|  |  | open | 0.027 | 0.007 |  |  |  |
|  |  | woody | 0.025 | 0.007 | 16.645 | >0.001 | *** |
| Turnover |  | exclosure | 0.175 | 0.009 |  |  |  |
|  |  | open | 0.013 | 0.012 |  |  |  |
|  |  | woody | 0.011 | 0.012 | 1.465 | 0.481 |  |
| Nestedness |  | exclosure | 0.023 | 0.006 |  |  |  |
|  |  | open | 0.012 | 0.008 |  |  |  |
|  |  | woody | 0.013 | 0.008 | 3.417 | 0.181 |  |

| **Table S2.3. Differences in seasonal beta diversity within site types**. Linear mixed-effects models were fitted by REML (restricted maximum likelihood). χ² and p-values are calculated from comparing nested models (with and without site type) via likelihood ratio tests. | | | | | | | |
| --- | --- | --- | --- | --- | --- | --- | --- |
| **Beta diversity metrics** |  | **Site type** | **Estimate** | **SE** | **X²** | **p-value** | |
| Sørensen |  | exclosure | 0.376 | 0.013 |  |  |  |
|  |  | open | 0.01 | 0.019 |  |  |  |
|  |  | woody | -0.004 | 0.019 | 0.603 | 0.74 |  |
| Turnover |  | exclosure | 0.308 | 0.015 |  |  |  |
|  |  | open | -0.006 | 0.021 |  |  |  |
|  |  | woody | -0.01 | 0.021 | 0.245 | 0.885 |  |
| Nestedness |  | exclosure | 0.068 | 0.011 |  |  |  |
|  |  | open | 0.016 | 0.015 |  |  |  |
|  |  | woody | 0.006 | 0.015 | 1.193 | 0.551 |  |

| **Table S2.4. Differences in seasonal beta diversity between site types.** Linear mixed-effects models were fitted by REML (restricted maximum likelihood). χ² and p-values are calculated from comparing nested models (with and without season) via likelihood ratio tests. | | | | | | | |
| --- | --- | --- | --- | --- | --- | --- | --- |
| **Beta metrics** | **Site type** | **Season** | **Estimate** | **SE** | **X²** | **p-value** | |
| Sørensen | exclosure Δ open | dry | 0.319 | 0.017 |  |  |  |
|  |  | wet | 0.036 | 0.024 | 2.273 | 0.132 |  |
|  | exclosure Δ woody | dry | 0.269 | 0.014 |  |  |  |
|  |  | wet | 0.05 | 0.019 | 6.54 | 0.011 | * |
|  | open Δ woody | dry | 0.281 | 0.014 |  |  |  |
|  |  | wet | 0.051 | 0.02 | 6.676 | 0.01 | ** |
| Turnover | exclosure Δ open | dry | 0.206 | 0.019 |  |  |  |
|  |  | wet | 0.077 | 0.027 | 7.976 | 0.005 | ** |
|  | exclosure Δ woody | dry | 0.189 | 0.016 |  |  |  |
|  |  | wet | 0.045 | 0.023 | 3.694 | 0.055 | . |
|  | open Δ woody | dry | 0.211 | 0.016 |  |  |  |
|  |  | wet | 0.072 | 0.022 | 9.69 | 0.002 | ** |
| Nestedness | exclosure Δ open | dry | 0.114 | 0.014 |  |  |  |
|  |  | wet | -0.042 | 0.019 | 4.643 | 0.031 | * |
|  | exclosure Δ woody | dry | 0.08 | 0.012 |  |  |  |
|  |  | wet | 0.005 | 0.017 | 0.095 | 0.758 |  |
|  | open Δ woody | dry | 0.071 | 0.01 |  |  |  |
|  |  | wet | -0.02 | 0.014 | 2.207 | 0.137 |  |
